# Early-onset extrahepatic cholangiocarcinoma in a young adult with a resected congenital biliary cyst: a rare case report

**DOI:** 10.1097/MS9.0000000000005280

**Published:** 2026-06-25

**Authors:** Munawar Hraib, Bashar Alkasem, Lana Mahasny, Ali Al Mawrdi

**Affiliations:** a Department of Oncology, Al-Bairuni University Hospital, Damascus, Syria; b Faculty of Medicine, Damascus University, Damascus, Syria; c Department of Gastroenterology, National University Hospital, Damascus, Syria; d Department of Internal Medicine, National University Hospital, Damascus, Syria

**Keywords:** biliary-enteric anastomosis, cholangiocarcinoma, choledochal cyst, early-onset, portal hypertension

## Abstract

**Introduction:**

Congenital choledochal cysts are well-recognized risk factors for subsequent hepatobiliary malignancy, particularly cholangiocarcinoma, even after surgical excision. However, the development of extrahepatic cholangiocarcinoma (eCCA) in early adulthood following neonatal resection of a congenital biliary cyst is exceptionally rare. We report a case of early-onset eCCA in a young woman who was previously treated surgically in the neonatal period.

**Case presentation:**

A 25-year-old woman with a history of neonatal resection of a congenital biliary cyst, cholecystectomy, and biliary-enteric jejunal anastomosis presented with progressive epigastric and left upper quadrant pain, postprandial vomiting, and marked weight loss. She had no jaundice, pruritus, or fever. At the age of 23, she had undergone further intervention for biliary stones, including endoscopic retrograde cholangiopancreatography, surgical stone extraction, and revision of the biliary-enteric anastomosis. Laboratory evaluation showed pancytopenia, microcytic anemia, preserved liver biochemistry, and elevated CA19-9. Magnetic resonance cholangiopancreatography demonstrated marked intrahepatic biliary dilatation with filling defects. Contrast-enhanced computed tomography revealed a large infiltrative upper abdominal mass invading the gastric wall and extending into the left hepatic lobe, with encasement of major vessels, portal hypertension, splenomegaly, ascites, and residual hilar biliary dilatation. Upper gastrointestinal (GI) endoscopy and endoscopic ultrasound showed extensive varices and an ulcerated gastric wall lesion caused by an external infiltrative mass. Endoscopic ultrasound-guided fine-needle aspiration demonstrated poorly differentiated invasive adenocarcinoma. Immunohistochemistry showed CK7 positivity and CK20 negativity, supporting a pancreatobiliary or upper GI origin. In the context of the patient’s surgical history and clinicoradiologic findings, the tumor was considered most consistent with unresectable eCCA. The patient was started on systemic chemotherapy with oxaliplatin and 5-fluorouracil.

**Discussion:**

This case illustrates several unusual and clinically important features. First, cholangiocarcinoma occurred at an exceptionally young age despite neonatal surgical treatment of the congenital biliary cyst. Second, the tumor presented without jaundice or biochemical cholestasis despite advanced local disease. Third, the lesion radiologically and endoscopically mimicked a primary gastric malignancy, adding diagnostic complexity. The case supports the concept that malignant transformation may occur decades after apparently corrective surgery, possibly due to persistent epithelial injury, residual biliary abnormalities, and chronic inflammatory changes related to prior biliary-enteric reconstruction.

**Conclusion:**

Early-onset eCCA after childhood resection of a congenital biliary cyst is exceptionally rare but possible. This case highlights the persistent, lifelong malignant potential associated with choledochal cysts and emphasizes that advanced disease may develop in the absence of jaundice or abnormal liver biochemistry. Long-term and possibly lifelong surveillance should be considered, particularly in patients with residual biliary abnormalities or prior biliary-enteric reconstruction.

## Introduction

Extrahepatic cholangiocarcinoma (eCCA) comprises perihilar and distal cholangiocarcinoma, both of which arise from anatomically distinct segments of the biliary tree^[^1^]^. Congenital choledochal cysts are recognized as a significant risk factor for the development of hepatobiliary malignancies, particularly eCCA, even after surgical resection^[^2^]^. Proposed mechanisms include chronic inflammation, prolonged pancreaticobiliary reflux, remnant epithelial abnormalities, and metachronous carcinogenesis^[^2,3^]^.

We report a rare case of early-onset eCCA in a 25-year-old woman who had undergone neonatal excision of a congenital biliary cyst. This case is notable for the unusually young age at presentation, the long latency period after surgical correction, the absence of jaundice, and an infiltrative mass with radiologic and endoscopic features mimicking a primary gastric malignancy. This report highlights the persistent malignant potential of choledochal cysts despite early surgical treatment.

## Case presentation

A 25-year-old woman with no history of smoking or alcohol use presented in mid-2025 with progressively worsening left upper quadrant and epigastric pain, severe enough to awaken her from sleep, exacerbated by food intake, and associated with postprandial vomiting. The course was accompanied by significant unintentional weight loss. There was no history of jaundice, pruritus, or fever. On examination, there was no jaundice, and no stigmata of chronic liver disease were documented.

### Past medical and surgical history

Neonatal period (8 days old): Resection of a congenital biliary cyst attached to the gallbladder, with cholecystectomy and biliary-enteric (jejunal) anastomosis.

Age 23: Episode of biliary colic. Imaging demonstrated intrahepatic duct dilatation with bile duct stones. She underwent endoscopic retrograde cholangiopancreatography, followed by surgical stone extraction and revision of the biliary-enteric anastomosis.

### Family and perinatal history

The patient was born to consanguineous parents. Maternal history was notable for severe hyperemesis gravidarum with weight loss. The family history included two first-trimester miscarriages and an infant death at 5 months of age due to a congenital cardiac anomaly.

### Laboratory findings

Initial laboratory evaluation revealed pancytopenia and microcytic anemia:

White blood cell: 2.3 × 10^9^/L (neutrophils/lymphocytes: 31.1%/61.2%)

Hemoglobin: 9 g/dL, Hematocrit: 27.9%

Red blood cell: 3.59 × 10^12^/L

Mean corpuscular volume: 77.7 fL, MCH: 25.3 pg

Platelets: 96 × 10^9^/L

Liver biochemistry showed no cholestasis or hepatocellular injury:

Alanine aminotransferase: 8 U/L, Aspartate aminotransferase: 15 U/L, Alkaline phosphatase: 123 U/L

Total bilirubin: 0.55 mg/dL, Direct bilirubin: 0.15 mg/dL

Albumin: 3.7 g/dL, Total protein: 6.5 g/dL

Renal function was preserved (urea: 10 mg/dL, creatinine: 0.5 mg/dL).

Coagulation profile showed mild prolongation (prothrombin time 15.6 s, International Normalized Ratio 1.2, partial thromboplastin time 36.2 s).

Inflammatory markers were elevated (erythrocyte sedimentation rate 40 mm/h, C-reactive protein 1.5 mg/dL).

Amylase was low-normal (16 U/L), lactate dehydrogenase was mildly elevated (222 U/L), and serum CA19-9 was elevated (415 U/mL).

HIGHLIGHTS

- Extrahepatic cholangiocarcinoma may occur in very young adults after neonatal choledochal cyst excision.
- Malignant transformation can occur decades after apparently corrective surgery.
- Advanced disease may present without jaundice or biochemical cholestasis.
- Lifelong surveillance should be considered after choledochal cyst resection, especially in high-risk patients.

### Diagnostic timeline

#### Magnetic resonance cholangiopancreatography (MRCP) – 4 September 2025

Marked intrahepatic biliary dilatation, predominantly involving the left system, with intraluminal low-signal filling defects compatible with biliary debris or stones. No pancreatic mass was identified. The common bile duct was not visualized, consistent with prior surgical reconstruction (Fig. 1).

**Figure 1.**
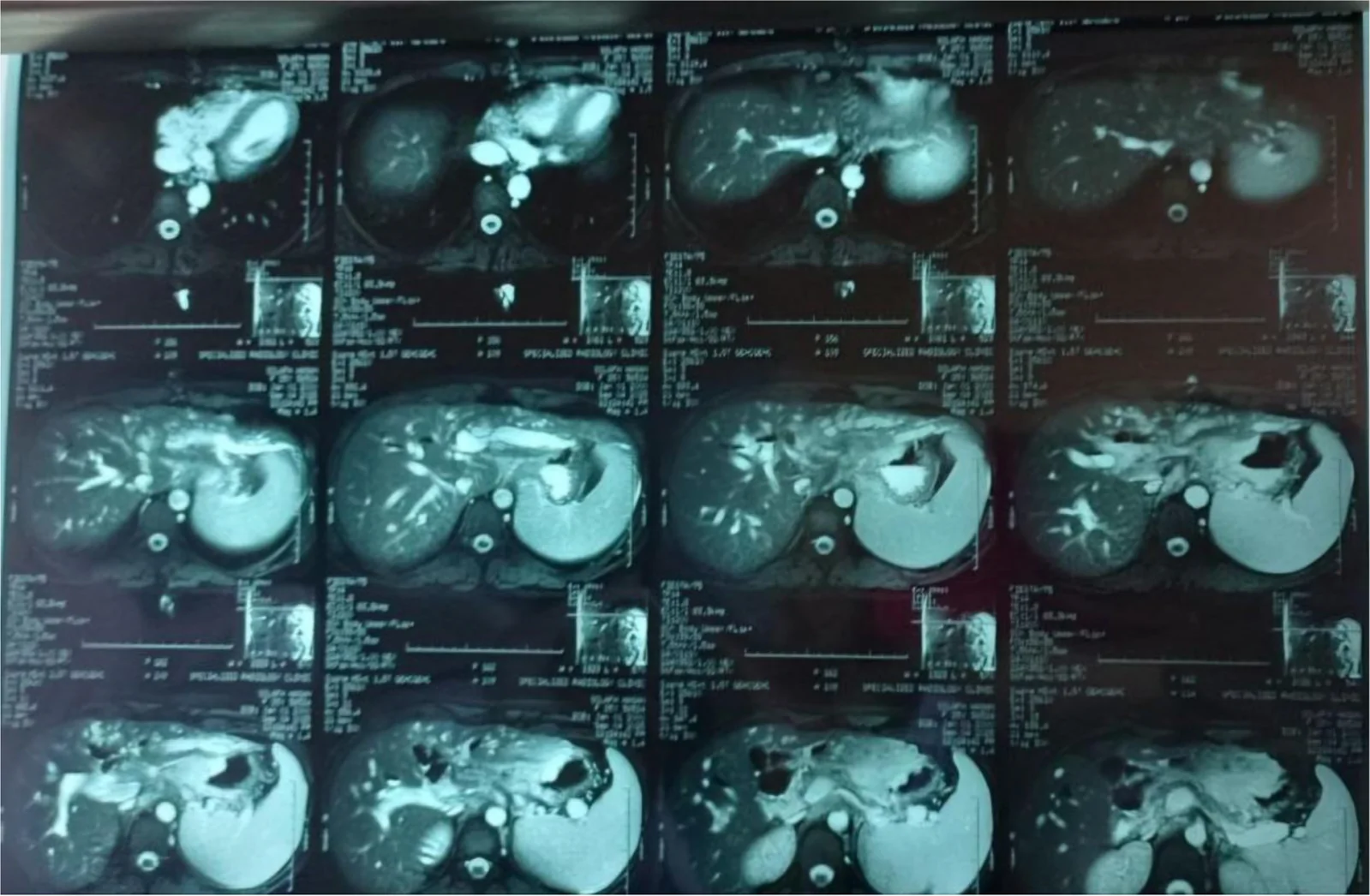
MRCP showing marked intrahepatic biliary ductal dilatation, more prominent in the left hepatic lobe, in a patient with a prior Roux-en-Y hepaticojejunostomy performed for a choledochal cyst.

#### Contrast-enhanced CT – 29 October 2025

A heterogeneous soft-tissue mass measuring approximately 60 × 80 mm was identified anterior to the stomach, with invasion of the gastric body and antrum, and contiguous extension into the left hepatic lobe. There was encasement and narrowing of the celiac axis, hepatic artery, splenic artery, and portal vein. Imaging demonstrated features of portal hypertension, including extensive perigastric and paraesophageal varices, and splenomegaly. A large lobulated cystic structure at the hepatic hilum (≈99 × 45 mm) was consistent with residual biliary dilatation. Moderate ascites were present (Fig. 2).

**Figure 2.**
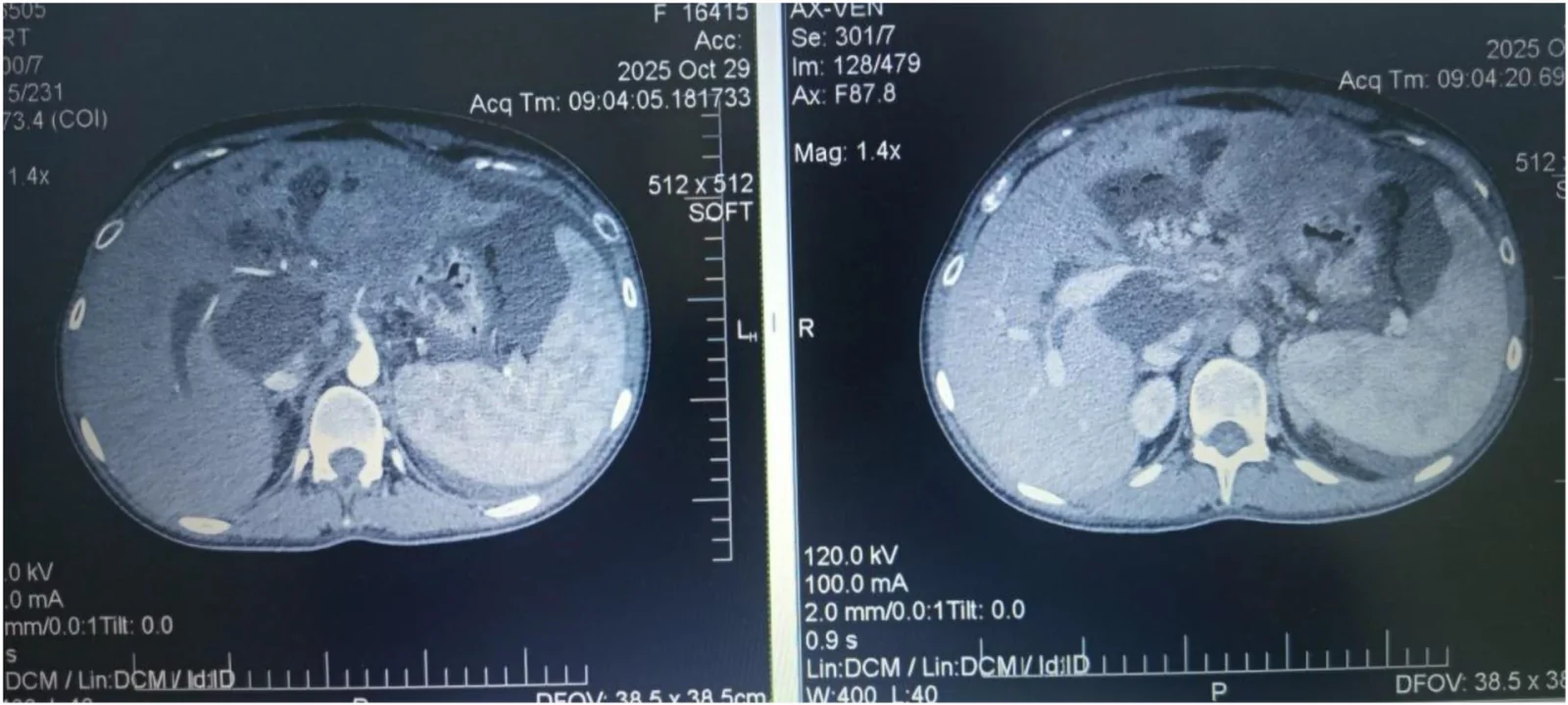
Contrast-enhanced axial CT images obtained in the arterial and portal venous phases. The images demonstrate a large, infiltrative upper abdominal mass centered in the gastric region and abutting the left hepatic lobe. There is soft-tissue encasement of the celiac axis with narrowing of the common hepatic and splenic arteries, which remain patent. Portal venous phase imaging shows occlusion of the main portal vein at its formation, with multiple periportal collateral vessels consistent with cavernous transformation (portal cavernoma). Associated splenomegaly is also noted.

#### Upper GI endoscopy – 11 November 2025

Large esophageal varices without stigmata of recent bleeding, a fundal varix, portal hypertensive gastropathy, and a broad ulcerated lesion on the anterior gastric wall at the junction of the body and antrum.

#### Endoscopic ultrasound (EUS) – 24 november 2025

Prominent esophageal, fundal, and duodenal varices were noted, confirming significant portal hypertension. A large hypoechoic mass (~50 × 52 mm) was visualized external to the pancreas and stomach, exhibiting an infiltrative pattern with penetration of the gastric wall layers up to the mucosa. Visualization of the portal vein lumen was limited due to extensive collateral circulation.

EUS-guided fine-needle aspiration revealed poorly differentiated invasive adenocarcinoma (Fig. 3).

**Figure 3.**
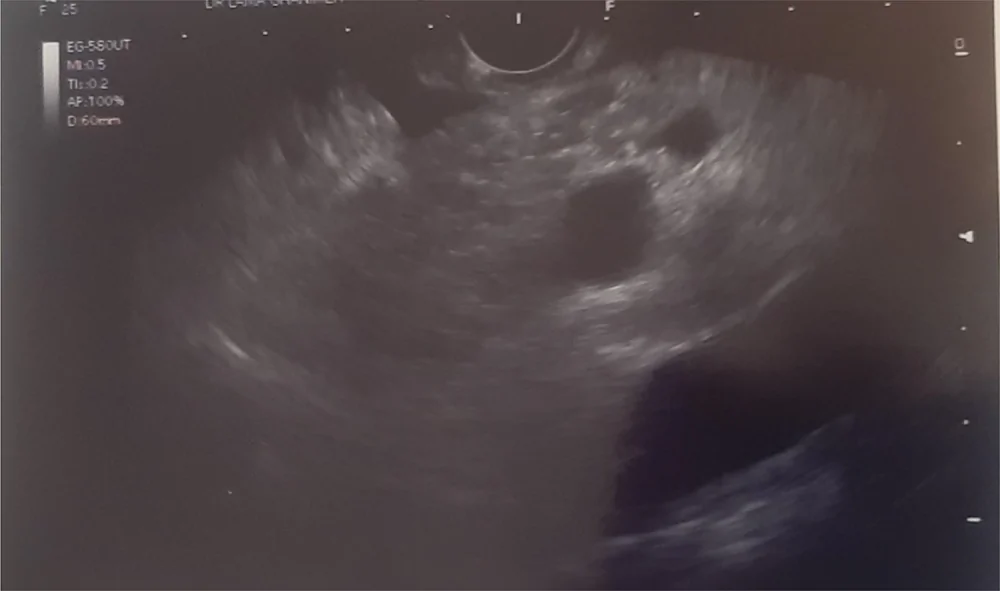
EUS demonstrates a large, irregular, hypoechoic mass external to the pancreas and stomach, with infiltrative features and apparent involvement of the gastric wall. Extensive collateral circulation/varices limited the clear delineation of the portal vein. EUS-guided fine-needle aspiration confirmed poorly differentiated invasive adenocarcinoma.

Immunohistochemistry showed CK7 positivity and CK20 negativity, supporting an upper gastrointestinal (GI) or biliary tract origin (Fig. 4).

**Figure 4.**
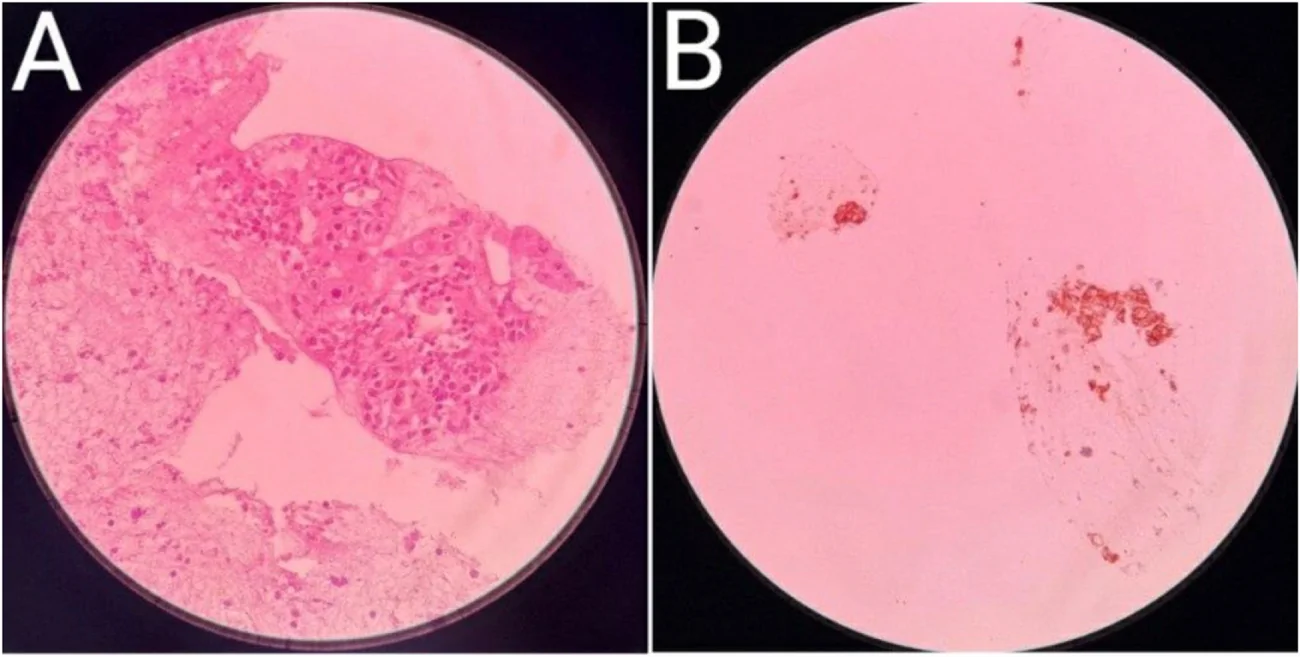
(A) Hematoxylin and eosin-stained section from the EUS-guided fine-needle aspiration specimen, demonstrating infiltrative atypical gland-forming epithelial cells consistent with poorly differentiated invasive adenocarcinoma. (B) Immunohistochemical staining showing CK7 positivity in tumor cells. The immunophenotype supports an upper gastrointestinal or biliary tract primary.

#### Final diagnosis and management

Given the patient’s history of a congenital biliary cyst with prior biliary-enteric reconstruction, the tumor was clinically favored to represent eCCA. The disease was deemed unresectable due to extensive vascular involvement and portal hypertension. The patient was initiated on systemic chemotherapy with oxaliplatin and 5-fluorouracil for advanced biliary tract carcinoma and remains under oncologic follow-up.

## Discussion

Cholangiocarcinoma is typically a malignancy of older adults, with incidence increasing with age, a slight male predominance, and peak diagnosis occurring between 50 and 70 years of age^[^4^]^. The development of cholangiocarcinoma in patients with congenital choledochal cysts reflects a distinct pathogenic pathway associated with a markedly increased lifetime risk of hepatobiliary malignancy, reported to range from 2.5% to 28%^[^5^]^. The underlying mechanism of carcinogenesis is thought to involve chronic epithelial injury caused by the abnormal reciprocal reflux of pancreatic juice and bile, resulting in repeated cycles of inflammation, mucosal damage, and regeneration^[^6^]^. Over time, this regenerative epithelium may undergo metaplastic and dysplastic transformation, accompanied by cellular atypia and molecular alterations such as K-ras and p53 mutations, ultimately promoting cholangiocarcinogenesis^[^6^]^. Although complete excision of the extrahepatic bile duct with bilioenteric reconstruction reduces this risk by separating bile from pancreatic secretions, it does not eliminate it completely; the residual risk remains higher than that of the general population, particularly in patients with persistent dilated intrahepatic ducts that may constitute a premalignant field^[^6,7^]^. In addition, postoperative factors such as reflux of activated pancreatic juice and bacterial contamination may further sustain chronic inflammation and ongoing carcinogenic processes^[^8^]^.

Choledochal cysts predominantly involve the large bile ducts, particularly the extrahepatic and perihilar segments, which are lined by mucin-producing cholangiocytes and contain peribiliary glands. These anatomical and cellular characteristics, together with the effects of pancreaticobiliary reflux, likely contribute to the stronger association with extrahepatic rather than intrahepatic cholangiocarcinoma^[^9^]^.

The present case demonstrated several atypical clinical features that contributed to diagnostic complexity. Notably, the patient presented without jaundice or biochemical evidence of cholestasis, despite advanced disease. This is in contrast to the classical presentation of eCCA, which is typically associated with obstructive jaundice^[^1^]^. In addition, the infiltrative mass exhibited radiologic and endoscopic features mimicking a primary gastric malignancy, further obscuring the diagnosis. These findings highlight that cholangiocarcinoma may present with non-specific or misleading clinical and imaging features, leading to potential delays in diagnosis^[^10^]^. The portal hypertension observed in this case is best explained by tumor encasement and narrowing of the portal vein, with subsequent development of collateral circulation, varices, and splenomegaly. The associated pancytopenia is most likely secondary to hypersplenism, a recognized consequence of portal hypertension that results in sequestration and increased destruction of blood cells within the enlarged spleen.

Immunohistochemically, cholangiocarcinoma typically expresses biliary epithelial markers such as CK7 and CK19, while CK20 expression is variable and often absent. However, this immunophenotype is not entirely specific, as overlapping staining patterns may also be seen in other upper GI adenocarcinomas^[^11,12^]^. Accordingly, immunohistochemistry should be interpreted in conjunction with the clinical, radiologic, and pathologic context rather than in isolation.

The CK7-positive/CK20-negative immunoprofile and elevated CA19-9 supported, but were not specific for, a pancreatobiliary origin. Notably, the gastric mucosa surrounding the lesion was preserved, and the tumor did not appear to arise from the gastric mucosa itself, arguing against a primary gastric carcinoma. Rather, the lesion appeared to originate outside the stomach, with secondary invasion of the gastric wall. In the context of the patient’s history of congenital choledochal cyst and prior biliary-enteric reconstruction, these findings favored eCCA.

This case carries important clinical implications. It underscores the persistent and potentially lifelong risk of malignancy in patients with choledochal cysts, even after early surgical excision. It highlights the limitations of relying solely on symptoms or liver biochemistry during surveillance, as advanced disease may develop in the absence of jaundice or biochemical cholestasis. Periodic imaging may, therefore, be particularly important in high-risk patients, especially those with residual biliary dilatation or prior biliary-enteric reconstruction^[^13^]^. A limitation of this report is the absence of a resection specimen and a broader immunohistochemical panel, making the assignment of the primary tumor site dependent on integrated clinicoradiologic–pathologic correlation.

## Conclusion

Early-onset eCCA after childhood resection of a congenital choledochal cyst is exceptionally rare. This case highlights that malignant transformation may occur decades after apparently corrective surgery and may present without jaundice or biochemical cholestasis. Long-term and possibly lifelong surveillance should be considered in patients with choledochal cysts, particularly when residual biliary abnormalities or prior biliary-enteric reconstruction are present.

## Data Availability

All the relevant data have been included in the manuscript itself.
